# Metabolite Profiling, Biological and Molecular Analyses Validate the Nutraceutical Potential of Green Seaweed *Acrosiphonia orientalis* for Human Health

**DOI:** 10.3390/nu16081222

**Published:** 2024-04-19

**Authors:** Bhakti Tanna, Sonam Yadav, Manish Kumar Patel, Avinash Mishra

**Affiliations:** 1 Division of Biotechnology and Phycology, CSIR-Central Salt and Marine Chemicals Research Institute, Bhavnagar 364002, India; 2 Academy of Scientific and Innovative Research (AcSIR), Ghaziabad 201002, India; 3 Department of Postharvest Science of Fresh Produce, Agricultural Research Organization (ARO), Volcani Center, Rishon LeZion 7505101, Israel

**Keywords:** *Acrosiphonia*, antiproliferative activity, bioactivity, functional food, human health, metabolomics, nutraceutical, seaweeds

## Abstract

Seaweeds have proven to be nutrient-dense and are rich in antioxidants, like phenolics, flavonoids, and other essential metabolites that help to provide their medicinal benefits. Non-targeted metabolite profiling of the tropical green seaweed *Acrosiphonia orientalis* showed the presence of numerous groups of contents, including sugars, essential amino acids, and fatty acids. Targeted metabolite profiling using HPLC identified 17 amino acids. The extract exhibited a very low half-maximal effective concentration (EC_50_) dosage for HeLa and Huh-7 cell lines, indicating a high likelihood of anticancer properties. A significant positive correlation was found between biological activities, such as antioxidation, scavenging, and reducing power with the phenolic and flavonoid contents. The extract revealed augmentation of proliferation in selected cervical cells, as it upregulated p53 1.3-fold, and downregulated important cancerous genes such as Cas-3 and DNMT 12- and 8-fold, respectively. An approximate 55-fold downregulation was observed in selected hepatic cell lines. Microarray analysis of hepatic cells indicated 0.27% and 0.07% upregulation of coding and non-coding genes, respectively, and 0.41% and 0.13% downregulation of coding and non-coding genes, respectively. As a consequence, it can be said that *A. orientalis* has possible medicinal use, such as anticancer activity, and therefore may be an intriguing food component that has potential as a regular dietary supplement.

## 1. Introduction

Seaweed utilization is a worldwide multimillion-dollar industry [1]. Though the concept of functional food is very recent, seaweeds have been employed as functional foods and medicinal herbs in Asian countries from ancient times [2,3]. Seaweed contains important vitamins, flavonoids, fibers, and minerals, as well as polyunsaturated fatty acids (PUFA) and other key metabolites; therefore, it has been considered beneficial for utilization as food [4,5]. *Acrosiphonia orientalis* is a chlorophyte in the family *Ulotrichaceae*. In India, *A. orientalis* is mainly found in the low-to-mid-intertidal zone [6]. Previous research on *A*. *orientalis* has shown its relevance in a wide range of fields. Lipid-soluble extract of *A*. *orientalis* revealed excellent antibacterial activity against multidrug-resistant infections [7]. The polysaccharide extract of *A*. *orientalis* demonstrated in vivo antiviral activity against shrimp pathogens and white spot syndrome virus (WSSV) [8]. Ethanolic and methanolic extracts of *A. orientalis* showed higher antimicrobial activity against selected Gram-positive and Gram-negative bacteria than chloroform extract [9]. Furthermore, methanolic extract displayed antibacterial activity against oral bacteria and might thus be used in mouthwash and chewing gums for treatment or prevention of dental caries [10]. *A. orientalis* harvested from the Vishakhapatnam coast (India) yielded about 11 mg/g of flavonoids, and flavonoids have high antioxidant properties and have shown protective effects against cancer and vascular problems in humans, as well as being best known to enhance the effects of ascorbic acid [11]. Methanolic extract of *A. orientalis* was found to be rich in fatty acids and to protect shrimps from *Vibrio harveyi* and *V. alginolyticus* infections [12].

Free radicals are frequently formed within acceptable limits owing to ordinary metabolic processes, but when produced in excess, they have been shown to cause a variety of human diseases, such as cancer, atherosclerosis, heart disease, hypertension, and diabetes [13]. The human body has antioxidant mechanisms to prevent such adverse effects on DNA, protein, or other molecules produced by free radicals up to a limit [13]. Thus, intake of promising antioxidants from novel food sources such as seaweeds is recommended [4,14,15]. Most natural antioxidants, such as polyphenolic compounds, including flavonoids, have proven to be non-toxic and safe [16]. According to various studies, ingestion of some polyphenolic compounds has been proven in many studies to increase life expectancy, reduce the development of chronic diseases, such as cancer, obesity, and diabetes, and even improve endothelial function and lower blood pressure [16,17]. Seaweeds are a rich source of physiologically active compounds and renewable resources. Therefore, they may be considered rich natural sources of novel metabolites containing a variety of biological activities that can be used for various medicinal and functional purposes. Chemical compounds, specifically secondary metabolites, are mainly linked to biological activities. Thus, these biologically active compounds may help us to anticipate some traditional and conventional uses of medicinal plants [18]. Nonetheless, maximum utilization of seaweed can be achieved by exploring novel potential areas of their application by using modern analytical tools and techniques [19].

The potential use of 100 seaweeds as low-fat dietary supplements was reported, and the nutraceutical potential of *A. orientalis* was shown as this alga has a low lipid content with a high level of C18 PUFAs [20]. Low-fat meals high in PUFA-rich nutraceuticals are responsible for improving the quality of the human diet. Thus, *A. orientalis* has the potential to be used in functional food and/or nutraceutical applications. There is no report on the bio-potential of *A. orientalis*, including metabolite composition, different biological activities, anti-proliferative potential, and its molecular mode of action on human cell metabolic pathways. This is the first report on comprehensive metabolomics (untargeted and targeted metabolite profiling), biological activities, anti-proliferative activity on human cancer cell lines, and a possible molecular mode of action on different human metabolic pathways, which unveils the potential of *A. orientalis* to be used in functional food and nutraceutical applications.

## 2. Materials and Methods

### 2.1. Sample Collection

The tropical seaweed *Acrosiphonia orientalis* (J.Agardh) P.C.Silva grows abundantly during the winter season. The seaweed was collected from the Saurashtra coast (India) of the Arabian Sea (Okha: N 22°28′8.19″; E 69°04′8.24″) during the winter season (November-December; average water temperature 24–25 °C) [5]. The identification was performed by seaweed taxonomic experts, voucher specimens from the herbarium, and also using the location-specific Taxonomic Reference Book [21]. Epiphytes and debris were cleaned with seawater and transferred to the laboratory under cool conditions. Some fresh seaweed samples were stored at −80 °C, and some seaweed samples were dried and stored at room temperature for future analysis.

### 2.2. Non-Targeted Metabolite Profiling: Extraction and Identification of Metabolites

About 100 mg of fresh samples was ground into a fine powder using a mortar and pestle. Metabolites were extracted with ice-cold methanol (2 mL, 70%, *v*/*v*) and further analysis of extracted metabolites was conducted by liquid chromatography–mass spectrometry. The background of each spectrum was subtracted, and the data were smoothed and then centered using Mass Lynx software version 4.1 (Micromass, Waters, Milford, MA, USA). Peaks were identified by comparison with the online METLIN database.

Similar to the above method, about 100 mg fine powdered sample was extracted with 100% ice-cold methanol. Adonitol (0.2 mg/mL) was used as an internal reference for quantification. Samples were prepared and analyzed by a gas chromatography–mass spectrometry instrument (GC-MS, Shimadzu, Kyoto City, Japan; Modal GC-MS TQ8040) equipped with an autosampler (AOC-5000, Shimadzu, Japan) and flame ionization detection (FID)/capillary column [22]. Mass spectra were recorded, and metabolites were identified by comparison with the NIST library. The concentration was measured with respect to the internal reference peak area and expressed as μg/g fresh weight.

### 2.3. Targeted Metabolite Profiling: Amino Acid Profiling

Total protein was extracted using the TCA/acetone method, and extracted protein was further quantified by the Bradford method. Further processing of extracted protein was conducted as per a previously optimized method [5], and extracted amino acids were analyzed by high-performance liquid chromatography (HPLC; model 2996, Water Alliance, Leeuwarden, The Netherlands). The instrument was equipped with an auto-sampler, and a C18 column with a particle size of 5 μm, pore size of 100 Å, length of 150 mm, and internal diameter of 4.6 mm (Luna, Phenomenex, Torrance, CA, USA) was used.

### 2.4. Targeted Metabolomics: Phenolic and Flavonoid Compounds

The sample stored in dried form was powdered and then passed through a sieve (85 mm), and extraction was performed with 10 g of powder using aqueous methanol (500 mL, 70%, *v*/*v*). Following centrifugation (7000× *g* at 25 °C for 10 min), the supernatant was decanted, concentrated in a rotary evaporator (100–150 mbar at 37 °C), then lyophilized and stored at −20 °C until use [5]. 

A standard solution (1 mg mL^−1^ (in acetonitrile:water; 1:1; HPLC-grade) concentration) for possible phenolic and flavonoid compounds was prepared as per previously optimized methods [5,22]. Standard and sample (seaweed extract) solutions were filtered (0.45 μm PVDF-syringe), diluted (100 ppm), and analyzed using an optimized HPLC method [5]. 

### 2.5. Estimation of Total Phenolic and Total Flavonoid Contents

Total phenolic (TPC) and flavonoid (TFC) contents were determined using standards (gallic acid and quercetin; respectively) [23]. TPC and TFC were calculated as μg mL^−1^ gallic acid and μg mL^−1^ quercetin per mg of extract, respectively [22].

### 2.6. Biological Activities

Different biological activities, including the total antioxidant, radical scavenging, reducing, and anti-proliferative activities of *A. orientalis* were measured [22]. Total antioxidant activity was expressed as a percentage inhibition of ABTS (2,2′-azino-bis(3-ethylbenzothiazoline-6-sulphonic acid)) and radical scavenging activity as a percentage inhibition of DPPH (2,2-diphenyl-1-picrylhydrazyl) free radicals [22,23]. The reducing capacity of the extract was calculated using ascorbic acid as a standard.

For the proliferation inhibition assay, widely used human cervical cancer cells (HeLa) and human hepatoma cancer cells (Huh-7) were selected and maintained. The assay was performed using the MTT-based in vitro Toxicology Assay Kit (Sigma-Aldrich, St. Louis, MO, USA) at 570 nm (690 nm for the blank).

### 2.7. Measurement of Intracellular ROS 

The intracellularly generated reactive oxygen species (ROS) superoxide anion (O_2_^−^) value was measured by a colorimetric nitroblue tetrazolium (NBT)-based assay [24]. The absorbance of the solution was read at 620 nm, and the percentage ROS inhibition activity was determined from the amount of formazan crystals generated.

### 2.8. Nuclear Staining with Hoechst Dye 33342 and DNA Fragmentation Study 

*A. orientalis* extract (EC_50_)-treated cells (Hela and Huh-7) were incubated with dye (Hoechst 33342) for 10 min at 37 °C and visualized under a fluorescence microscope (Olympus, Tokyo, Japan) to evaluate staining of apoptotic cells [25]. For the DNA fragmentation study, genomic DNA was isolated from treated and untreated cells, and about 1 μg DNA was electrophoresed (1% agarose gel), stained with ethidium bromide, and analyzed under UV light [25].

### 2.9. Transcript Expression Analysis by Quantitative Real-Time PCR

Total RNA was extracted from cells (treated with EC_50_ of *A. orientalis* extract) and converted into cDNA using reverse transcriptase. Quantitative real-time PCR (qRT-PCR) was performed to evaluate the transcript concentration of key cancer-related genes, such as *Cas-3* (encoding Caspase-3), *p53* (encoding tumor suppressor protein p53), *CDC2* (encoding cell division cycle protein 2), *DNMT* (encoding DNA methyl transferase), and *BAX* (encoding BCL2-associated X, an apoptosis regulator), whereas the commonly used housekeeping gene *GAPDH* (encoding glyceraldehyde-3-phosphate dehydrogenase enzyme) was used as an internal reference gene [26]. qRT-PCR was performed with SYBR green using optimized conditions and gene-specific primers (Supplementary Table S1). The relative expression (2^−ΔΔCt^) was compared between treated and non-treated (control) cells [27]. 

### 2.10. Microarray Based Differential Gene Expression Analysis

Total RNA was isolated from cells (HeLa and Huh-7; treated and untreated), and converted to first-strand cDNA, from which second-strand cDNA was synthesized, followed by cRNA and finally, single-stranded cDNA [28]. Single-stranded cDNA was fragmented and labeled according to the manufacturer’s user manual (Affymetrix, Santa Clara, CA, USA). The labeled cDNA was hybridized at 42 °C for 16 h with a GeneChip Human Transcriptome Array 2.0 (HTA 2.0). Hybridized chips were washed and stained using a fluidics module (GeneChip Fluidics Station 450, Affymetrix, USA) as per the manufacturer’s instructions. Chips were scanned (Scanner 3000 7G; Affymetrix, USA), and differential analysis was performed with transcriptome analysis console software (Version 4.0.2, Thermo Fisher Scientific Inc., Waltham, MA, USA).

### 2.11. Data Mining and Statistical Analysis

All bioactivity and biochemical experiments were performed three times, and each experiment was performed in triplicate. Analysis of variance (ANOVA) and Tukey’s honest significant difference (HSD) were used in combination to evaluate between- and within-group variation. The threshold for significance was taken as *p* < 0.05, and all values were expressed in terms of mean ± standard error of the mean (SE), with statistically significant differences indicated by letters between means. All biochemical and biological activities were subjected to correlation and multivariate analysis using regression analysis, principal component analysis (PCA), and Pearson’s correlation matrix. MetaboAnalyst ver. 5.0 was used for metabolic network analysis and pathway enrichment analysis [29]. Metabolite concentrations were normalized by median for sample normalization, log10 transformed for data transformation, and auto-scaled to the mean for data scaling.

## 3. Results

### 3.1. Metabolite Profiling (Non-Targeted)

Various primary and secondary metabolites or their derivatives belonging to fatty acids, phospholipids, sugars, phenolic compounds, flavonoids, and anthocyanins were observed in *A. orientalis* by non-targeted metabolite profiling by LC-MS and GC-MS. About 24 and 4 different metabolites were detected in −ve and +ve modes, respectively, in the range 110.048–1080.54 *m*/*z* with different additives by LC-MS. Most metabolites showed a resemblance to flavonoids or lipids (Supplementary Table S2). The metabolites (primary and secondary) were also determined by non-targeted metabolite profiling by GC-MS. A total of 47 metabolites or their derivatives belonging to different groups, such as amino acids, sugars, fatty acids, and their derivatives were identified (Table 1). Among the sugars, sucrose (1830 µg g^−1^ Dw) was estimated at the highest concentration, followed by maltose (1430 µg g^−1^ Dw). Some tricarboxylic acid (TCA) cycle metabolites were found in *A. orientalis*. Malic acid (175 µg g^−1^ Dw) was found at the highest concentration, followed by citric acid (5 µg g^−1^ Dw) and succinic acid (4 µg g^−1^ Dw). Fatty acids are also important metabolites identified in *A. orientalis.* Palmitic acid (57 µg g^−1^ Dw) and stearic acid (50 µg g^−1^ Dw) showed the greatest increase in *A. orientalis.* The seaweed *A. orientalis* was also subjected to targeted metabolite profiling to detect the amino acids and flavonoids precisely.

### 3.2. Targeted Metabolite Profiling

A total of 17 amino acids was detected in *A. orientalis* by HPLC, out of which 8 were essential, 6 were conditionally essential, and 3 were other amino acids (Table 2). In total, 128 ± 1 µg mg^−1^ Dw (dry weight) amino acids was detected, of which 75 ± 1 µg mg^−1^ Dw was essential amino acids (EAA), 50 ± 2 µg mg^−1^ Dw was conditional EAA, and 3 ± 0.5 µg mg^−1^ Dw was other amino acids. Among EAA, phenylalanine was detected in the maximum concentration (50 ± 2 µg mg^−1^ Dw) followed by leucine (9 ± 0.5 µg mg^−1^ Dw) and lysine (8 ± 2 µg mg^−1^ Dw). The conditional EAA tyrosine was detected as the most abundant amino acid (40 ± 10 µg mg^−1^ Dw), followed by another amino acid, aspartate (17 ± 1 µg mg^−1^ Dw). 

The seaweed *A. orientalis* was found to be rich in total phenolic and flavonoid contents, detected as 196.27 ± 15.72 μg mL^−1^ GAE (gallic acid equivalents) per mg extract and 376.015 ± 47.58 μg mL^−1^ quercetin equivalent per mg extract, respectively. The presence of the identified phenolic and flavonoid compounds was detected in *A. orientalis* by targeted metabolite profiling using HPLC (Table 3). The flavonoid catechin (20 µg mg^−1^ Dw) was found at the highest concentration, followed by ascorbic acid (10 µg mg^−1^ Dw), gallic acid (7 µg mg^−1^ Dw), and protocatechuic acid (6 µg mg^−1^ Dw). Other key flavonoids, curcumin, myricetin, and rutin hydrate, were detected in the range of 0.1–0.2 µg mg^−1^ Dw. The flavonoids rutinoside, sinapic acid, naringenin, nobiletin, apigenin, kaempferol, coumarin, and luteolin were detected at 0.01–0.05 µg mg^−1^ Dw, and the lowest concentration was observed for p-Coumaric acid (0.002 µg mg^−1^ Dw).

### 3.3. Biological Activities

**Figure 1:**
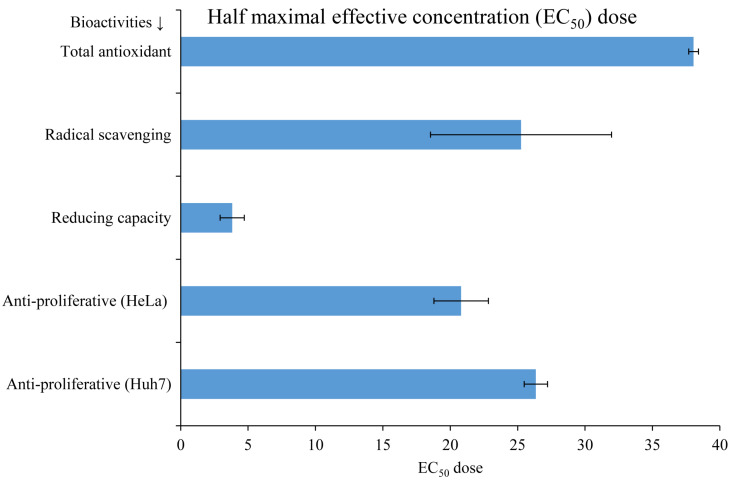
Determination of the half-maximal effective concentration (EC_50_) of *Acrosiphonia orientalis* for different bioactivities. Different concentrations of extracts were tested, EC_50_ was determined, and expressed as mean ± SE (*n* = 3).

The total antioxidant (ABTS), scavenging (DPPH), and reducing activities of *A. orientalis* extract were found to be dose-dependent (Supplementary Figure S1). At a lower dose (20 µg), scavenging activity was higher, although antioxidant activity was higher at higher doses. In comparison, reducing activity was higher than both antioxidant and scavenging activities (Supplementary Figure S1B). The half-maximal effective concentration (EC_50_) of *A. orientalis* extract was found to be highest at about 38.05 mg Dw for antioxidant activity, followed by anti-proliferative activity for cancerous cell lines (Huh-7: 26.35 mg Dw, and HeLa: 20.81 mg Dw), scavenging (25.25 mg Dw), and reducing activity (3.82 mg Dw) (Figure 1 and Supplementary Table S3). A lower EC_50_ dosage validated the potential bioactivities of *A. orientalis* and the possibilities of its exploration as a dietary supplement, natural antioxidant, and anticancer functional food.

Correlation analysis showed that total antioxidant, scavenging, and reducing activities were correlated with total phenolic and flavonoid contents (Supplementary Table S4). Total antioxidant (ABTS inhibition) activity showed a significant (*p* < 0.05) and very strong correlation with TPC (0.916, R^2^ = 0.839), TFC (0.983, R^2^ = 0.966), and scavenging (DPPH) activity (0.913, R^2^ = 0.833), and a strong but nonsignificant (*p =* 0.57) correlation with the reducing capacity (0.867, R^2^ = 0.752) of the *A. orientalis* extract. Similarly, scavenging activity (DPPH) was very strongly and significantly (*p* < 0.005) correlated with TPC (0.975, R^2^ = 0.951) and TFC (0.969, R^2^ = 0.939) while showing a nonsignificant (*p =* 0.25) moderate correlation with the reducing capacity (0.630, R^2^ = 0.397) of the *A. orientalis* extract. The reducing capacity of the *A. orientalis* extract showed a strong correlation with TFC (0.775, R^2^ = 0.601) and a moderate correlation with TPC (0.670, R^2^ = 0.449), but this correlation was not significant (*p* > 0.1). A very strong (0.949, R^2^ = 0.901) and significant (*p* < 0.05) correlation was observed between TPC and TFC. All of these correlations were positive, revealing that the TPC and TFC of *A. orientalis* had a significant impact on its applicability as a functional food.

### 3.4. Metabolic Network and Pathway Enrichment Analysis to Elucidate the Interactions between Metabolite Changes and Metabolic Pathways

**Figure 2:**
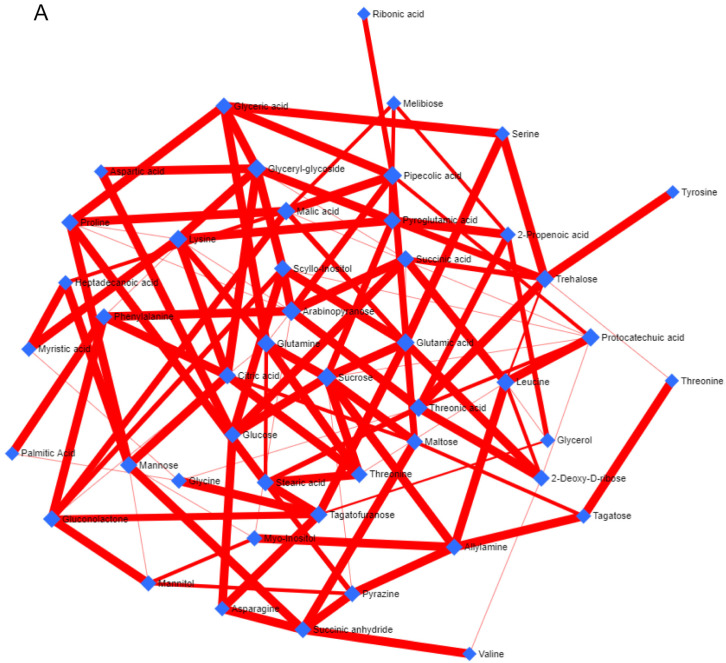

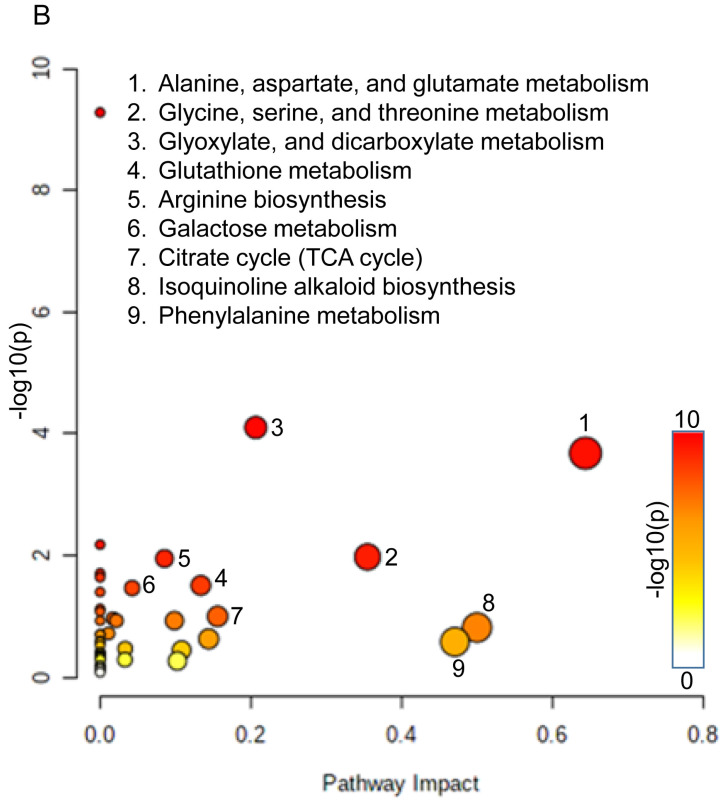
Network and pathways enrichment analysis of *A*. *orientalis* by GC-MS. (**A**) The *A*. *orientalis* GC-MS dataset was utilized to build a metabolites–metabolites network. Nodes and edges represent the metabolites and their correlations, respectively. Nodes indicate distinct metabolites, while edges represent different levels of correlation between different metabolites. Positive correlations are shown by red edges. The thicker edge represents a stronger correlation between metabolites, whereas the thinner edge represents a weaker correlation. (**B**) The *A*. *orientalis* dataset was used for the pathway enrichment analysis, which depicted metabolic networks. The results from the pathway enrichment analysis are explained in detail in Table S5.

**Figure 3:**
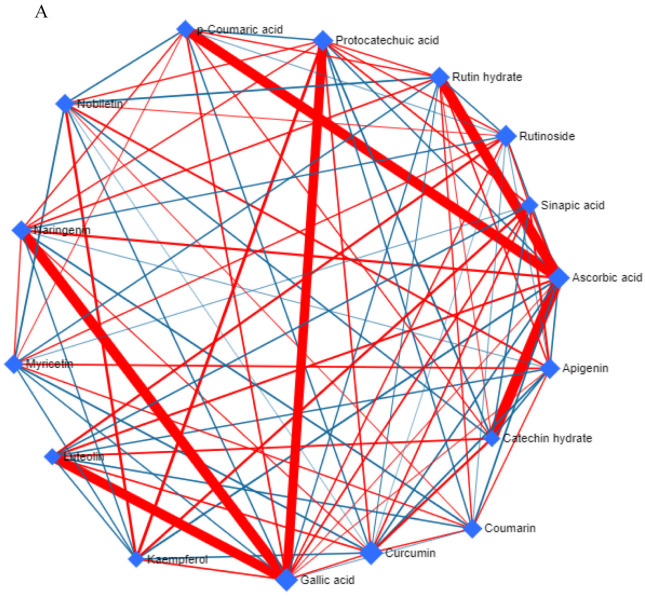

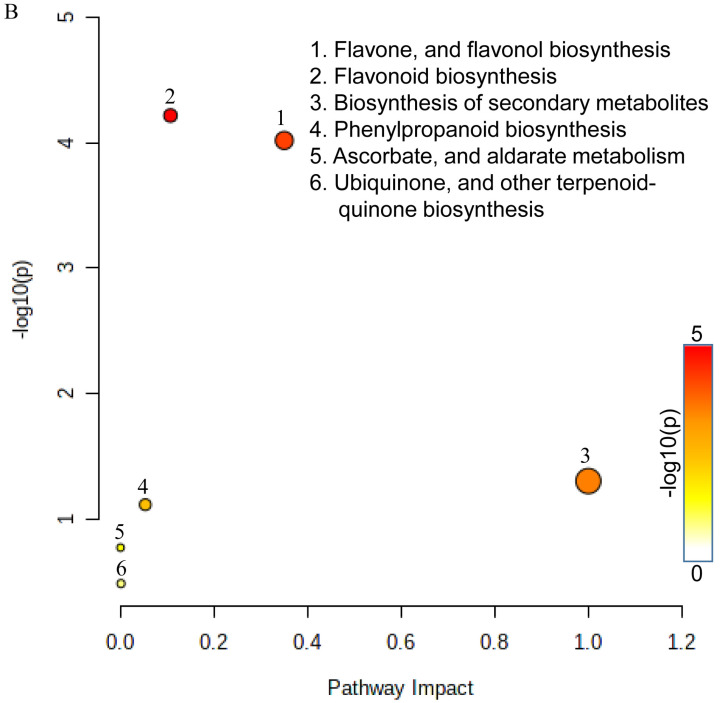
Network and pathways enrichment analysis of *A*. *orientalis*. (**A**) The HPLC dataset of *A*. *orientalis* was utilized to build a metabolites–metabolites correlation network. The metabolites and their associations are represented by nodes and edges, respectively. Different colored edges indicate different levels of correlation between distinct metabolites, while nodes represent metabolites. Positive correlations are shown by red edges, whereas negative correlations are represented by blue edges. The thicker edge indicates a stronger correlation between metabolites, whereas the thinner edge indicates a lesser correlation. (**B**) Graphical illustration of pathway enrichment analysis showing metabolic networks from the dataset of *A*. *orientalis*. The results from the pathway enrichment analysis are explained in detail in Table S6.

A metabolite–metabolite correlation-based network analysis was performed to understand the interrelationships of different metabolites in *A*. *orientalis* using GC-MS and HPLC datasets (Figure 2 and Figure 3). The metabolite–metabolite correlation was constructed through the range of *p*-values using the debiased sparse partial correlation (DSPC) with different network properties, such as node neighbors, average node degree, betweenness filter, degree filter, and correlation filter (Figure 2A and Figure 3A). The ideal thresholds for the current investigation were determined by a degree filter cutoff of 2.0 and a betweenness filter cutoff of 1.0. Positive correlations are shown as red edges, whereas negative correlations are represented by blue edges; thicker and medium red edges indicate stronger and weaker positive correlations between the metabolites, respectively. The network constructed from the *A*. *orientalis* GC-MS dataset included 46 nodes representing the identified metabolites and 118 edges suggesting strong positive correlations and variations in their interaction (Figure 2A). Sucrose had strong positive correlations with eight (glucose, maltose, glutamine, glutamic acid, pyroglutamic acid, threonine, and allylamine) different metabolites (Figure 2A). Furthermore, the network generated from the *A*. *orientalis* HPLC dataset had 16 nodes representing the metabolites and 91 edges demonstrating positive and negative correlations and variations in their interaction (Figure 3A). The metabolites–metabolites correlation might be positive or negative based on the content of metabolites in *A*. *orientalis* (Figure 2A and Figure 3A). Furthermore, the significantly altered metabolites were mapped onto the KEGG reference pathway of *Arabidopsis thaliana* in order to correlate the detected metabolites with the corresponding biological pathways that are active in *A*. *orientalis* (Figure 2B and Figure 3B). For pathway analysis, various parameters were employed, including the visualization method (scatter plot), enrichment method (hypergeometric test), and topology analysis (relative-betweenness centrality). The metabolic pathways are shown as a circle based on their score from enrichment analysis (−log10(p)) and topology analysis (pathway impact) (Figure 2B and Figure 3B). The brighter red color and larger circle size represent the higher metabolite alterations in the relevant pathway and pathway impact value, respectively (Figure 2B and Figure 3B). In this study, 47 and 6 metabolic pathways were identified from *A*. *orientalis* metabolites using GC-MS (Table 1) and HPLC (Table 3) datasets, respectively (Supplementary Tables S5 and S6). In the analysis performed with the GC-MS metabolite dataset, seven metabolic pathways (alanine, aspartate, and glutamate metabolism; glycine, serine, and threonine metabolism; glyoxylate and dicarboxylate metabolism; glutathione metabolism; citrate cycle (TCA cycle); phenylalanine metabolism; and isoquinoline alkaloid biosynthesis) were found to have higher impact values (≥0.1) in *A*. *orientalis*, whereas arginine biosynthesis and galactose metabolism had low impact values (Figure 2B). Furthermore, in an analysis performed on the HPLC dataset, three metabolic pathways (flavonoid biosynthesis, flavone, and flavonol biosynthesis, and biosynthesis of secondary metabolites—unclassified) were found to have greater impact values (≥0.1) in *A*. *orientalis* (Figure 3B). However, the impact values of phenylpropanoid biosynthesis and ubiquinone and other terpenoid–quinone biosyntheses were low.

### 3.5. Apoptosis Analysis and ROS Inhibitory Activity

**Figure 4:**
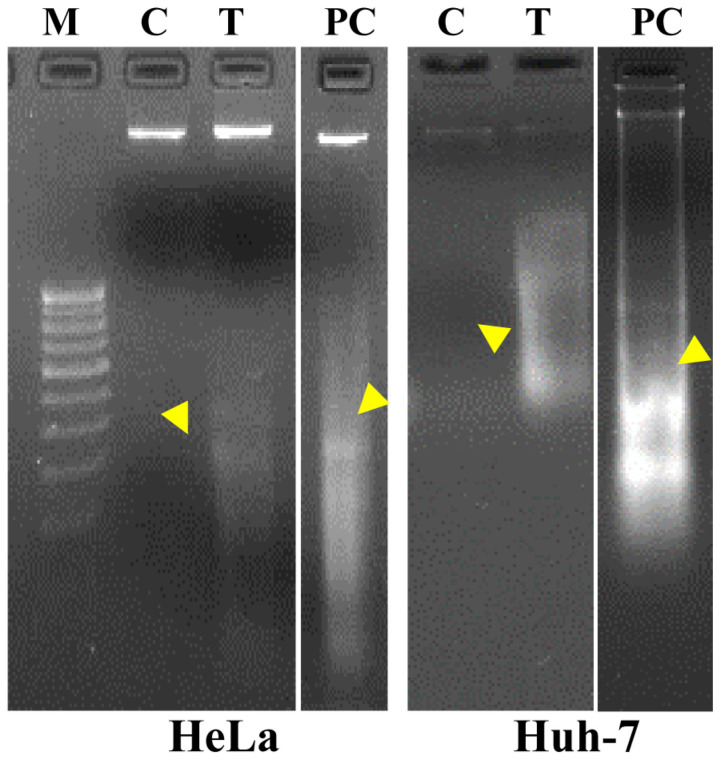
Apoptosis analysis by DNA fragmentation. Agarose gel (1%) showed DNA fragmentation of cells (HeLa and Huh-7) treated with an EC_50_ dose of *Acrosiphonia orientalis* extracts. M: Marker (100 bp DNA ladder), C: control (untreated cells), T: treated cells, PC: positive control (cells treated with DMSO). Yellow color triangle shows DNA fragmentation.

**Figure 5:**
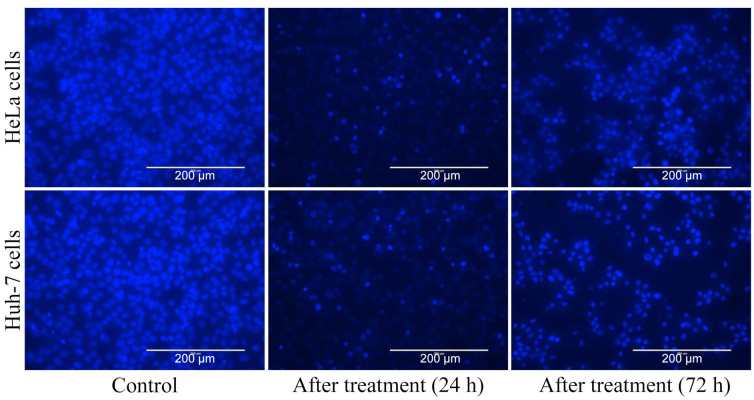
Apoptosis analysis by Hoechst assay. The DNA-specific fluorescent dye (Hoechst 33342) was used to stain cells (HeLa and Huh-7) treated with an EC_50_ dose of seaweed (*Acrosiphonia orientalis*) extracts for 24 h and 72 h, and observed under florescent microscope. Control was untreated cells.

DNA fragmentation is a marker of the apoptosis process, which was investigated using DNA fragmentation (Figure 4) and nuclear staining (Figure 5). Agarose gel electrophoresis clearly showed DNA fragmentation in cancerous cells (HeLa and Huh-7) treated with an EC_50_ dose of *A. orientalis* extract (Figure 4) compared to control. Hoechst 33342 is a DNA-specific fluorescent dye that binds to fragmented DNA, and hence it was used to study chromatic condensation and/or nuclear fragmentation. DNA-specific staining of cancerous cells showed DNA fragmentation (Figure 5) as observed in agarose gel electrophoresis. In comparison with control cells, cells treated with an EC_50_ dose of *A. orientalis* extract exhibited strong fluorescence, indicating strong binding. Cancer cell lines were also used in the ROS-inhibitory assay. It was observed that an EC_50_ dose of *A. orientalis* extract inhibited ROS generation by 40% in the cell lines within 24 h (Supplementary Figure S2). Overall, the study showed the potential of *A. orientalis* extract to inhibit ROS as well as cancer cell apoptosis. 

### 3.6. Transcript Expression Analysis by Quantitative Real-Time PCR

**Figure 6:**
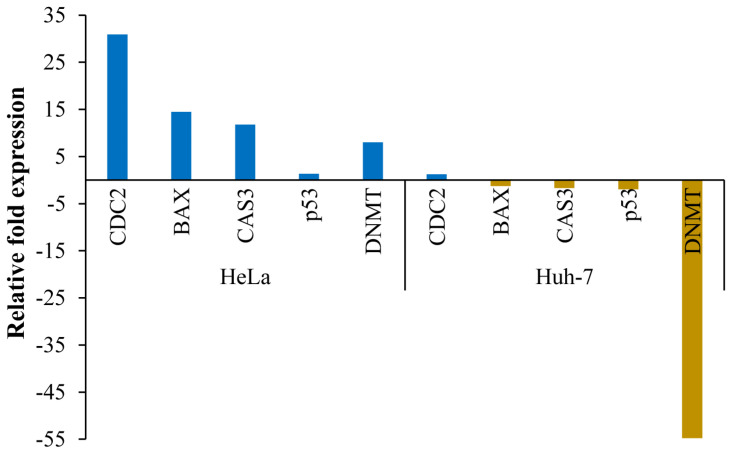
Transcript expression analysis of key cancer-linked genes. Expression is analyzed by quantitative real-time PCR and the relative expression (2^−ΔΔCt^) of genes cells treated with *Acrosiphonia orientalis* extract were compared with control (untreated cells). Gene *GAPDH* is used as internal reference.

The transcript expression of key cancer-linked genes (*CDC2*, *BAX*, *CAS3*, *p53*, and *DNMT*) was studied in the cancer cell lines HeLa and Huh-7 (Figure 6). All genes were upregulated in HeLa cells but downregulated (except *CDC2*) in Huh-7 cells treated with an EC_50_ dose of *A. orientalis* extract. The *CDC2* gene, which encodes cell division cycle protein 2, was upregulated by a maximum of about 30-fold followed by 15-fold upregulation of the apoptosis regulator B-cell lymphoma 2 (BCL2)-associated X protein encoding gene *BAX*. Caspase-3 encoding gene *Cas3* and the DNA methyl transferase encoding gene *DNMT2* were upregulated by about 12- and 8-fold, respectively, in treated HeLa cells. The tumor suppressor gene *p53*, which encodes protein p53, was also slightly (1.3-fold) upregulated in treated HeLa cells. Interestingly, all key genes (except *CDC2*) were downregulated in Huh-7 cells, with the *DNMT* gene being the most downregulated, by 55-fold. The results indicated that *A. orientalis* extract prevented the proliferation of hepatoma cancer cells more strongly than that of cervical cancer cells. 

### 3.7. Microarray-Based Differential Gene Expression Analysis

Human transcriptome array (HTA) chips were used for microarray analysis. The HTA chips are a high-resolution array with unique probes that cover both coding and non-coding transcripts. Exons for coding transcripts are covered by about 70% of the probes, whereas the remaining 30% cover non-coding transcripts and/or exon-exon splice junctions.

Differential gene expression analysis was performed, and gene levels showing a fold change <−2 or >2 with *p* < 0.05 were considered in the analysis. A probe-set (gene/exon) was considered expressed if ≥50% of the samples had threshold DABG (detection above background) values. Scattered and volcano plots confirmed that out of a total of 67,528 transcripts (including both coding and non-coding), 350 transcripts (0.52%) were differentially expressed by <−2 or >2 -fold change with *p* < 0.05 in Huh-7 cells, treated with an EC_50_ dose of *A. orientalis* compared to untreated (control) cells (Supplementary Figure S3 and Supplementary Table S7). About 138 (39.43%) were upregulated, and 212 (60.57%) transcripts were downregulated. Among upregulated transcripts, 88.41% were coding transcripts, while 11.59% were non-coding. Similarly, among downregulated transcripts, 85.85% were coding, while 14.15% were non-coding genes (Supplementary Figure S4). 

Out of 44,699 coding sequences, 304 were differentially expressed (<−2 or >2), of which 0.27% (122) were upregulated, 0.41% (182) were downregulated, and the remaining 99.32% were unchanged. Similarly, out of 22,829 non-coding probes, 46 were differentially expressed (<−2 or >2), out of which 0.07% (16) were upregulated, 0.13% (30) were downregulated and the remaining 99.8% remained unchanged (Supplementary Figure S4). Differentially expressed genes were distributed across all the chromosomes (Supplementary Figure S5). 

Out of a total of 67,528 transcripts, 19,472 transcripts (28.84%) passed the filter criteria of the exon splicing index (>2 or <−2 with *p* < 0.05). Out of a total of 44,699 coding sequences, 17,810 (91.46%) had a differential exon splicing index, while out of 22,829 non-coding genes, 1662 (28.84%) had a differential exon splicing index. Among 19,742 transcripts showing a differential exon splicing index, 4876 showed all splicing events, 1669 showed a complex event, 1314 an alternative last exon, 958 an alternative first exon, 289 an alternative 5′ donor site, 277 intron retention, 181 a cassette exon, 179 an alternative 3′ acceptor site, and 9 mutually exclusive exons (Supplementary Figure S6).

**Figure 7:**
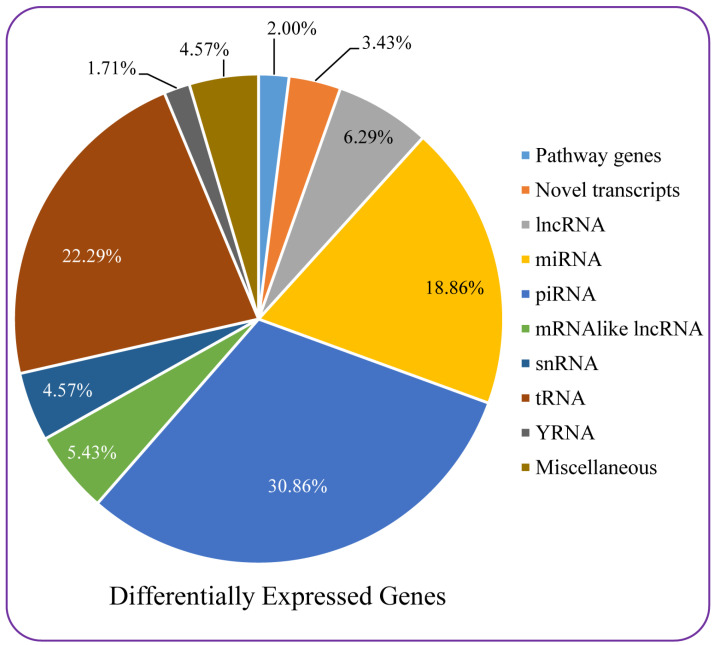
Differentially expressed genes analyzed by microarray. Genes differentially expressed in Huh-7 cells treated with EC_50_ dose of *Acrosiphonia orientalis* extract compared with control (untreated cells) belonged to different groups.

Among 350 differentially expressed transcripts, most transcripts (30.86%) were piRNAs (piwi-interacting RNAs), followed by 22.29% tRNAs (transfer RNAs), 18.86% miRNAs (micro RNAs), 6.29% lncRNAs (long non-coding RNAs), 5.43% mRNA, such as lncRNAs, and 4.57% snRNA (small nuclear RNAs). About 3.43% of differentially expressed transcripts were novel transcripts, and 2% genes belonged to different functional pathways. The remaining 4.57% transcripts were miscellaneous (Figure 7). Seven key functional genes were found that were differentially expressed (up or down) in Huh-7 cells (Table 4). 

Gene ontology enrichment analyses of differentially expressed transcripts confirmed significant alteration of a total of 22 pathways in Huh-7 cells treated with an EC_50_ dose of *A. orientalis* compared to untreated (control) cells. Out of these 22 pathways, the tumor suppressor pathway was highly influenced, and downregulation of miRNA involved in the tumor pathway was observed. Downregulation of miRNA663B modulated suppression of the TP53 pathway. Similarly, glutamate binding, activation of AMPA receptors, synaptic plasticity, and G-protein signaling pathways were downregulated. Feline McDonough Sarcoma-like tyrosine kinase (FLT3), chemokine signaling, and MAPK signaling pathways were upregulated. By contrast, signaling through ROBO receptors was downregulated, and further analysis indicated that key pathways involved in proliferation were downregulated (Supplementary Table S7).

## 4. Discussion

*Acrosiphonia orientalis*, formally known as *Spongomorpha indica* L., is a medicinal green seaweed. It is distributed along intertidal regions of tropical and subtropical seawaters and originated from the parent plant *Spongomorpha kutzing* [9]. It is nutritionally rich in vitamin C and therefore widely utilized for medicinal purposes and as a dietary supplement [30]. Other green seaweeds, such as *Caulerpa* spp., have proven their nutraceutical potential by containing significant levels of phenolic and flavonoid compounds as well as high antioxidant and anti-proliferative activities on different cell lines [22]. 

In this study, a rarely studied or underutilized green seaweed with a strong therapeutic potential was investigated by metabolic profiling and biological activities. *A. orientalis* was thoroughly analyzed using a combination of metabolomics and molecular biology. The LC-MS data confirmed that *A. orientalis* is rich in flavonoid derivatives, such as catechin, epicatechin, quercetin, and apigenin derivatives, and could therefore be a potential antioxidant for human health [5]. Specifically, HPLC analysis employing standards confirmed the presence of catechin hydrate in very high quantities followed by ascorbic acid, gallic acid, and protocatechuic acid in significantly elevated amounts. The presence of a high amount of protocatechuic acid was also confirmed by GC-MS. Protocatechuic acid belongs to the complex polyphenols, which include anthocyanins, and has been shown to have antioxidant, anti-inflammatory, antiaging, anticancer, and even neuroprotective, anti-osteoporotic, antibacterial, and antiviral properties [31]. These compounds may be responsible for its exceptionally high reducing potential [22]. It has also been proven to contain all of the essential amino acids, such as phenylalanine, leucine, lysine, isoleucine, methionine, threonine, histidine, and valine, in descending order. Among these amino acids, leucine, isoleucine, and valine are branched-chain amino acids that help in the building of muscle proteins and reduction of muscle breakdown, resulting in anabolic responses in humans [32]. Tagatose was also shown to be present in significant quantities among the various sugars obtained. Tagatose is gaining importance as a nutraceutical due to its potential use in the food and feed industries [33]. Apart from that, it also has antidiabetic properties, a lower calorific value, and the ability to promote the growth of beneficial gut bacteria [33]. Malic acid was also found to be present in our seaweed. Malic acid is a weak acid present in numerous fruits, including pears and apples. It has been shown to have the capacity to produce numerous health benefits, such as regulation of pH, protection of the cardiovascular system, and inhibition of α-glucosidase activity [34]. Malic acid has been found to cure dry mouth in xerostomia patients and to enhance oral health-related quality of life [35]. Malic acid ingestion improved digestive traits in Japanese quails [36]. In summary, the presence of different metabolites detected by LC-MS, GC-MS, and HPLC exhibited the potential of *A. orientalis* to be utilized for several nutraceutical benefits.

The biological activities of our seaweed have been proven to be exceptional when compared with prior research on different seaweeds. *A. orientalis* showed very strong reducing power with an EC_50_ value of 5 μg mL^−1^, whereas in previous studies, the green seaweed *Caulerpa* spp. had shown an 86.4 ± 3.1 μg mL^−1^, which was the lowest value among all those of the collected green seaweeds [22]. In a study including red seaweeds, the lowest reducing power was reported for *Sarconema scinaioides*, which was 237 ± 3 μg mL^−1^, which is higher than our observation for *A. orientalis* [37]. This shows the exceptional activity of *A. orientalis*. It also showed significantly lower EC_50_ values for total antioxidant activity, radical-scavenging activity, and anti-proliferative activity on HeLa and Huh-7 cell lines, which were 40 ± 1, 25 ± 7, 20 ± 2, and 25 ± 1, respectively. Furthermore, as shown in the Supplementary Table S4 (except for the reducing power capacity with TPC and TFC), biological activities had a substantial positive correlation with phenolic and flavonoid contents. This observation is consistent with the fact that our seaweed had a high nutritional content, which was responsible for its high biological activities.

One of the main processes that is frequently used to assess the effectiveness of anticancer therapy is apoptosis. Apoptosis is a type of programmed cell death that occurs in cells to maintain homeostatic conditions in response to aging or stress. Apoptosis can be studied using various assays, such as DNA fragmentation, caspase detection, membrane potential, vacuole formation, etc. However, more than one assay is required to confirm the occurrence of apoptosis [38]. Thus, in our study, we performed more than one assay: in the DNA ladder assay, which evaluates DNA fragmentation using agarose gel electrophoresis, we observed bands of about 200 bp, indicating DNA fragments produced by endonuclease activation of cells due to apoptosis (Figure 2) [39], and in the Hoechst 33342 assay, fragmented DNA was found to have increased in quantity (Figure 3), and hence both the assays demonstrated that apoptosis was occurring. 

ROS can be a source of many pathological conditions, such as aging, cancer, and even cardiovascular diseases. Polyunsaturated fatty acids and cholesterol are peroxidized by ROS, which causes a reduction in membrane permeability. They also oxidize nucleic acid, which causes mutations and cancer. Hence, scavenging of ROS can play an important role in suppressing such ailments [25]. The NBT assay was used to investigate superoxide scavenging activity, and it was observed that in the presence of seaweed extract (EC_50_ dose), cancer cells produce less formazan, indicating the presence of fewer superoxide ions [24]. As a result, it can be said that *A. orientalis* extract has the capacity to suppress superoxide radicals. Consequently, it can be concluded that our extract may have anticancer and ROS-scavenging properties in the selected cell lines. The limitation of the study is that results were obtained under laboratory conditions using a methanolic extract, which does not accurately replicate the physiological conditions of both animals and humans. Factors, such as enzymatic digestion, pH variations, and microbial metabolism in the gut can significantly alter the bioavailability of nutrients [40]. 

Further, to examine the factors responsible at the molecular level, we performed gene expression studies using RT-PCR and microarrays. Caspases and other upstream regulatory factors, known as oncogenes or tumor suppressor genes, influence cell death during apoptosis by directing proteolytic activity [41]. In our study, 12-fold upregulation was seen in HeLa cells. p53 is a pro-apoptotic gene that controls growth and cancer propagation by encouraging apoptosis in cells with high DNA damage. As a defense mechanism, it retards cell growth, and promotes DNA repair or initiates apoptosis in cells with irreversible DNA damage [42]. In HeLa cells, p53 was shown to be upregulated 1.3-fold. DNA methyltransferases (DNMTs) regulate the DNA methylation process, which is a key factor in gene expression. Recent studies have suggested that defective DNMTs could lead to tumor progression [43]. The drugs that can act as DNMT inhibitors can be used as anticancer drugs. In our study, *A. orientalis* extract showed downregulation of DNMT up to 55-fold in Huh-7 cell lines, the highest degree of downregulation among all the other genes selected. It was observed that in Huh-7 cell lines, all the genes were downregulated (except CDC2), while in HeLa, all the genes were upregulated.

In Huh-7 cell lines, microarray analysis showed that 0.27% of coding genes and 0.07% of non-coding genes were upregulated while downregulation of 0.41% of coding genes and 0.13% of non-coding genes occurred. Several forms of cancer have been linked to aberrant expression of miRNA663B. In a study targeting bladder cancer cells, downregulation of miRNA663B prevented proliferation and triggers apoptosis. T24 cell viability was considerably decreased, and apoptosis was strongly triggered by miRNA663B suppression. Furthermore, miRNA663B suppression increased the p53 and p21 expression levels in T24 cells [44]. The polyphenol compound luteolin has been shown to exhibit antitumor activity by affecting miRNA663B expression [45]. In our study, miRNA663B was downregulated, which can be linked to the anticancer property of the extract. Another study showed the effect of downregulation of ROBO on cancer cells. Slit protein functions by attaching to a single-pass transmembrane protein (Robo). A study has shown that Slit2 dramatically reduces lung cancer cell migration, demonstrating suppression of lung cancer through Slit-Robo signaling [46]. 

Feline McDonough sarcoma (FMS)-like tyrosine kinase 3 (FLT3), a member of the class III receptor kinase family, is also known as fetal liver kinase-2, stem cell tyrosine kinase 1 (TK), or CD135. Deregulation of the FLT3 receptor plays a significant role in the development of leukemia and is often tightly limited to the early progenitor cells where it is expressed. Although its function in these tissues is uncertain, FLT3 is also found in tissues such as the placenta, brain, cerebellum, and gonads. A complex is created when the FLT3 receptor interacts with its ligand. The creation of this complex activates molecules involved in signal transduction, which spreads the signal throughout the cell. Cell proliferation, differentiation, and survival are influenced by FLT3 signaling [47]. Marine macrolides have been shown to be efficient inhibitors of tyrosine kinase 3 inhibitors in in silico virtual screening [48]. In our study, CD135 was found to be upregulated, which may be linked to the anticancer property of the extract. When the tumor develops and metastasizes, various immune-related substances (cytokines, chemokines, and immunosuppressive drugs) infiltrate the tumor environment. Hence, compounds that downregulate chemokine signaling can lead to a decrease in tumor progression. Various natural products have been shown to act on these types of pathways [49]. Fucoxanthin from brown seaweed has been shown to decrease chemokine receptor-4 (CXCR4), which is responsible for the migration, invasion, and adherence of cancer cells to endothelial cells, inhibiting in vivo metastases [50]. Fucoxanthin has also been shown to inhibit the MAPK pathway, which is responsible for cancer cell migration [51]. In our study, the extract was shown to decrease chemokine signaling, which indicates its antitumor properties. Hence, as per the differential gene expression study, it can be concluded that *A. orientalis* exerts antitumor activity through regulation of various pathways.

## 5. Conclusions

This study has demonstrated the nutraceutical potential of *A. orientalis*. There has been no previous study showcasing an extensive metabolite or gene-level analysis of this seaweed. Therefore, this study helps in providing considerable evidence on the beneficial effects of seaweed. Nevertheless, animal studies should be carried out to extrapolate the data from this study regarding high antioxidant and anticancer properties. In addition, in-depth analysis is still required to evaluate other activities occurring due to its high antioxidant properties.

## Figures and Tables

**Table 1:** Metabolites detected in *A. orientalis* by GC-MS.

Metabolites	Name	Amount ^
Amino acid	Asparagine	12 ± 7
Amino acid	Aspartic acid	8 ± 0.5
Amino acid	Glutamic acid	50 ± 18
Amino acid	Glutamine	2 ± 0.2
Amino acid	Glycine	7 ± 2
Amino acid	Leucine	4 ± 0.05
Amino acid	Lysine	15 ± 3
Amino acid	Phenylalanine	3 ± 0.5
Amino acid	Proline	1 ± 0.2
Amino acid	Serine	3 ± 0.1
Amino acid	Threonine	3 ± 0.02
Amino acid	Threonine	4 ± 1
Amino acid	Tyrosine	3 ± 0.3
Amino acid	Valine	6 ± 1
Amino acid derivative	Pyroglutamic acid	18 ± 8
Dicarboxylic acid	Succinic acid	4 ± 0.4
Fatty acid	Heptadecanoic acid	1 ± 0.3
Fatty acid	Myristic acid	6 ± 2
Fatty acid	Palmitic acid	57 ± 8
Fatty acid	Stearic acid	50 ± 8
Organic acid	2-Propenoic acid	13 ± 5
Organic compound	Allylamine	4 ± 1
Organic compound	Citric acid	5 ± 1
Organic compound	Malic acid	175 ± 5
Organic acid	Pipecolic acid	36 ± 3
Organic compound	Pyrazine	8 ± 4
Organic compound	Succinic anhydride	1 ± 0.1
Phenolic acid	Protocatechuic acid	630 ± 15
Polyol compound	Glyceryl-glycoside	24 ± 2
Sugar	2-Deoxy-D-ribose	10 ± 2
Sugar	Arabinopyranose	20 ± 4
Sugar	Glucose	2 ± 0.5
Sugar	Maltose	1430 ± 30
Sugar	Mannose	11 ± 2
Sugar	Melibiose	1 ± 0.01
Sugar	Myo-Inositol	12 ± 1
Sugar	Sucrose	1830 ± 145
Sugar	Tagatofuranose	4 ± 0.4
Sugar	Tagatose	100 ± 10
Sugar	Trehalose	80 ± 8
Sugar acid	Gluconolactone	13 ± 1
Sugar acid	Glyceric acid	5 ± 1
Sugar acid	Glycerol	8 ± 0.4
Sugar acid	Ribonic acid	6 ± 2
Sugar acid	Threonic acid	24 ± 8
Sugar alcohol	Mannitol	28 ± 2
Sugar alcohol	Scyllo-Inositol	18 ± 2

^ Amount (mean ± SE; *n* = 3) in µg g^−1^ Dw (dry weight). Values are rounded up as per significant certainty numbers.

**Table 2:** Amino acids content quantified in *A. orientalis* using HPLC.

Amino Acids	Amount ^
*Essential amino acids (EAA)*	
Histidine	0.3 ± 0.1
Isoleucine	4 ± 2
Leucine	9 ± 0.5
Lysine	8 ± 2
Methionine	1 ± 0.1
Phenylalanine	50 ± 2
Threonine	2 ± 0.1
Valine	0.01 ± 0.0
*Conditional EAA*	
Arginine	2 ± 0.2
Cysteine	5 ± 1
Glutamic acid	0.3 ± 0.05
Glycine	0.03 ± 0.00
Proline	2 ± 0.1
Tyrosine	40 ± 10
*Others amino acids*	
Alanine	0.3 ± 0.1
Aspartic acid	17 ± 1
Serine	2 ± 0.1

^ Amount (mean ± SE; *n* = 3) in µg g^−1^ Dw (dry weight). Values are rounded up as per significant certainty numbers.

**Table 3:** Metabolites quantified in *A. orientalis* using HPLC.

Metabolites	Amount ^
Apigenin	0.01
Ascorbic acid	10
Catechin hydrate	20
Coumarin	0.03
Curcumin	0.1
Gallic acid	7
Kaempferol	0.01
Luteolin	0.05
Myricetin	0.1
Naringenin	0.01
Nobiletin	0.01
p-Coumaric acid	0.002
Protocatechuic acid	6
Rutin hydrate	0.2
Rutinoside	0.01
Sinapic acid	0.01

^ Amount (mean ± SE; *n* = 3) in µg g^−1^ Dw (dry weight). Values are rounded up as per significant certainty numbers.

**Table 4:** Key functional genes that were differentially expressed (up- or downregulated) in Huh-7 cells treated with an EC_50_ dose of *A. orientalis* compared to untreated cells and analyzed by microarray using HTA2.0 microarray chip.

Microarray Gene Prob Id	Fold Change Log 2	Chromosome Number	Strand	Group	Gene Description/Role
TC10001334.hg.1	2.4	chr10	-	Coding	Annexin II
TC22000851.hg.1	2.07	chr22	-	Coding	ATP synthase, H+ transportation
TC0X001435.hg.1	−2.25	chrX	-	Coding	CDR1 gene encoding for cerebellar degeneration-related protein 1
TC04001578.hg.1	2.08	chr4	-	Coding	Protein phosphatase 1, regulatory (inhibitor) subunit
TC07002266.hg.1	−2.79	chr7	+	Non-coding	scaRNA (small Cajal body-specific RNAs)
TC05001641.hg.1	−2.12	chr5	-	Coding	Solute carrier organic anion transporter (SLCO4C1)
TC11000565.hg.1	−2.15	chr11	+	Coding	Transmembrane protein 179B (TMEM179B)

## Data Availability

All data are provided in the article and Supplementary Material.

## Supplementary Materials

The following supporting information can be downloaded at: https://www.mdpi.com/article/10.3390/nu16081222/s1, Table S1: Primer sets and optimized PCR conditions for quantitative real-time PCR [26]; Table S2: Key metabolites detected in LC-MS analysis (Metabolites detected in *A. orientalis* showing resemblance with existing metabolites from METLIN database); Table S3: Half-maximal effective concentration (EC_50_) dose of *Acrosiphonia orientalis* for different bioactivities; Table S4: Correlation matrix of different activities (total antioxidant, scavenging, and reducing) and phenolic (TPC) and flavonoid (TFC) contents of *Acrosiphonia orientalis*; Table S5: Pathway analysis results of log-transformed and range-scaled metabolite abundance by GC-MS in *A*. *orientalis*; Table S6: Pathway analysis results of log-transformed and range-scaled metabolite abundance by HPLC in *A*. *orientalis*; Table S7: Differentially expressed genes in Huh-7 cells analyzed by microarray using HTA2.0 gene chip; Figure S1: Biochemical activities of *Acrosiphonia orientalis* extract. Total antioxidant activity, scavenging activity, and reducing capacity of *Acrosiphonia orientalis* are measured as percentage inhibition and expressed as mean ± SE (*n* = 3). For total antioxidant and scavenging activities, 20–100 µg mL^−1^ dose, while for reducing capacity, 10–50 µg mL^−1^ dose used. (A) Different activities, (B) different dose applied; Figure S2: ROS inhibitory activity of *Acrosiphonia orientalis*. Activity is shown as percentage radical inhibition in the cell lines treated with EC_50_ dose of *Acrosiphonia orientalis* extract. Data are expressed as mean ± standard error of the mean (SE; *n* = 3); Figure S3: Scattered and volcano plots showing differentially expressed genes. Plots showing genes up- or downregulated (<–2 or >2) in Huh-7 cells treated with EC_50_ dose of *A. orientalis* compared to untreated (control) cells; Figure S4: Differentially expressed genes in Huh-7 cells. Microarray analysis showed total number of genes (A) upregulated, (B) downregulated, number of (C) coding genes and (D) non-coding genes differentially expressed (up or downregulated; <–2 or >2) in Huh-7 cells treated with EC_50_ dose of *Acrosiphonia orientalis* compared to untreated (control) cells; Figure S5: Differentially expressed genes in Huh-7 cells. Microarray analysis showed differentially expressed genes are distributed across the chromosomes; Figure S6: Differentially expressed genes showing exon splicing index.
