# Supplementary material for: Metabolite Profiling, Biological and Molecular Analyses Validate the Nutraceutical Potential of Green Seaweed Acrosiphonia orientalis for Human Health
Source: Nutrients. 2024 Apr 19;16(8):1222. doi: 10.3390/nu16081222 (PMC11055090; doi:10.3390/nu16081222)
Supplement: Supplementary file 1 [file nutrients-16-01222-s001.zip › Figures S1-S6.pptx]

## Slide 1
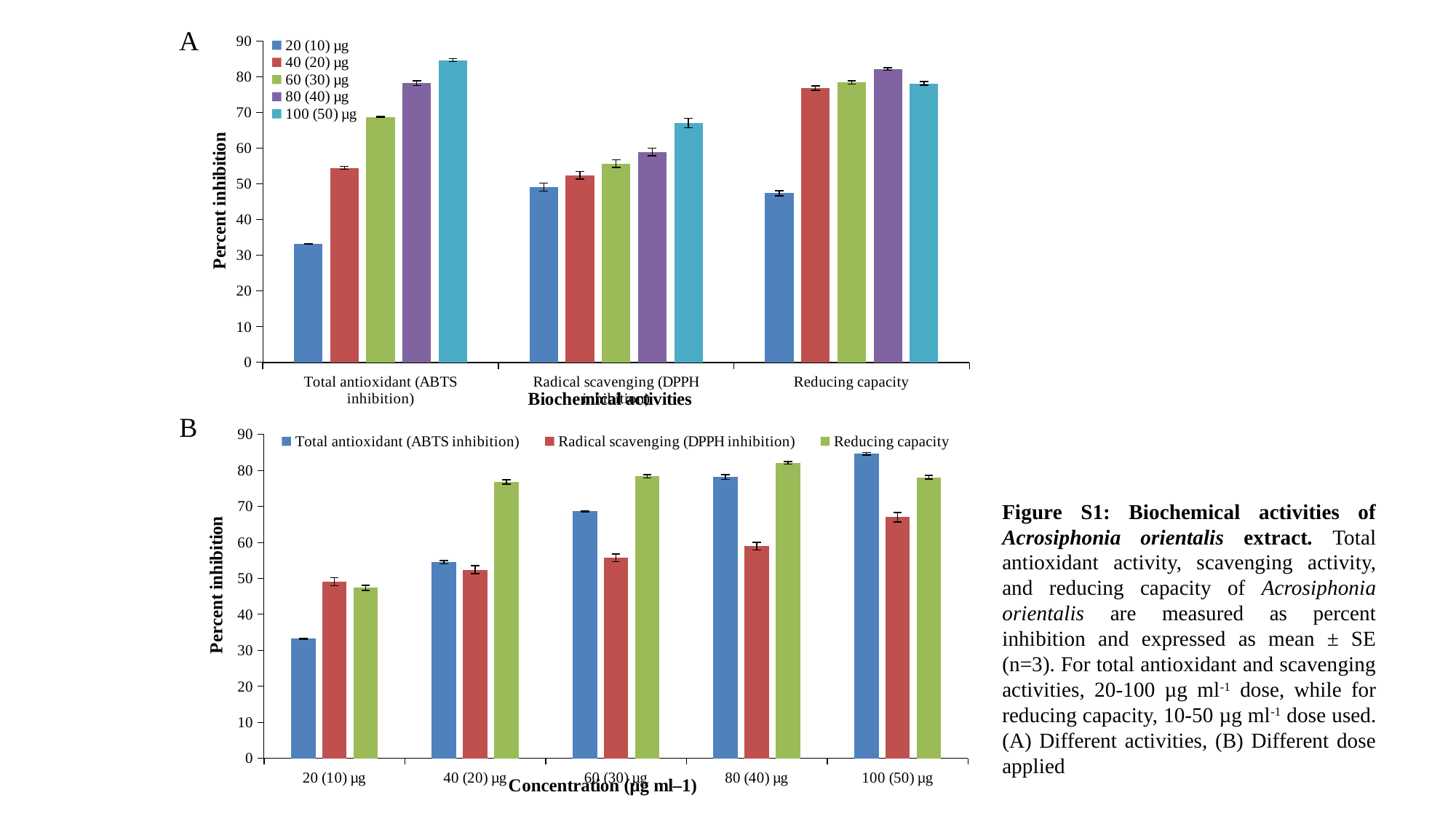

A
B
### Chart
| Category | 20 (10) µg | 40 (20) µg | 60 (30) µg | 80 (40) µg | 100 (50) µg |
|---|---|---|---|---|---|
| Total antioxidant (ABTS inhibition) | 33.243206591906 | 54.4951218014277 | 68.72137737736587 | 78.18284554353214 | 84.6717560817007 |
| Radical scavenging (DPPH inhibition) | 49.12866666666667 | 52.413333333333334 | 55.69800000000001 | 58.98266666666667 | 67.03319256906127 |
| Reducing capacity | 47.41175037297054 | 76.79542472994666 | 78.37400073748769 | 82.15910951949043 | 78.12935824643134 |
### Chart
| Category | Total antioxidant (ABTS inhibition) | Radical scavenging (DPPH inhibition) | Reducing capacity |
|---|---|---|---|
| 20 (10) µg | 33.243206591906 | 49.12866666666667 | 47.41175037297054 |
| 40 (20) µg | 54.4951218014277 | 52.413333333333334 | 76.79542472994666 |
| 60 (30) µg | 68.72137737736587 | 55.69800000000001 | 78.37400073748769 |
| 80 (40) µg | 78.18284554353214 | 58.98266666666667 | 82.15910951949043 |
| 100 (50) µg | 84.6717560817007 | 67.03319256906127 | 78.12935824643134 |Figure S1: Biochemical activities of Acrosiphonia orientalis extract. Total antioxidant activity, scavenging activity, and reducing capacity of Acrosiphonia orientalis are measured as percent inhibition and expressed as mean ± SE (n=3). For total antioxidant and scavenging activities, 20-100 µg ml-1 dose, while for reducing capacity, 10-50 µg ml-1 dose used. (A) Different activities, (B) Different dose applied

## Slide 2
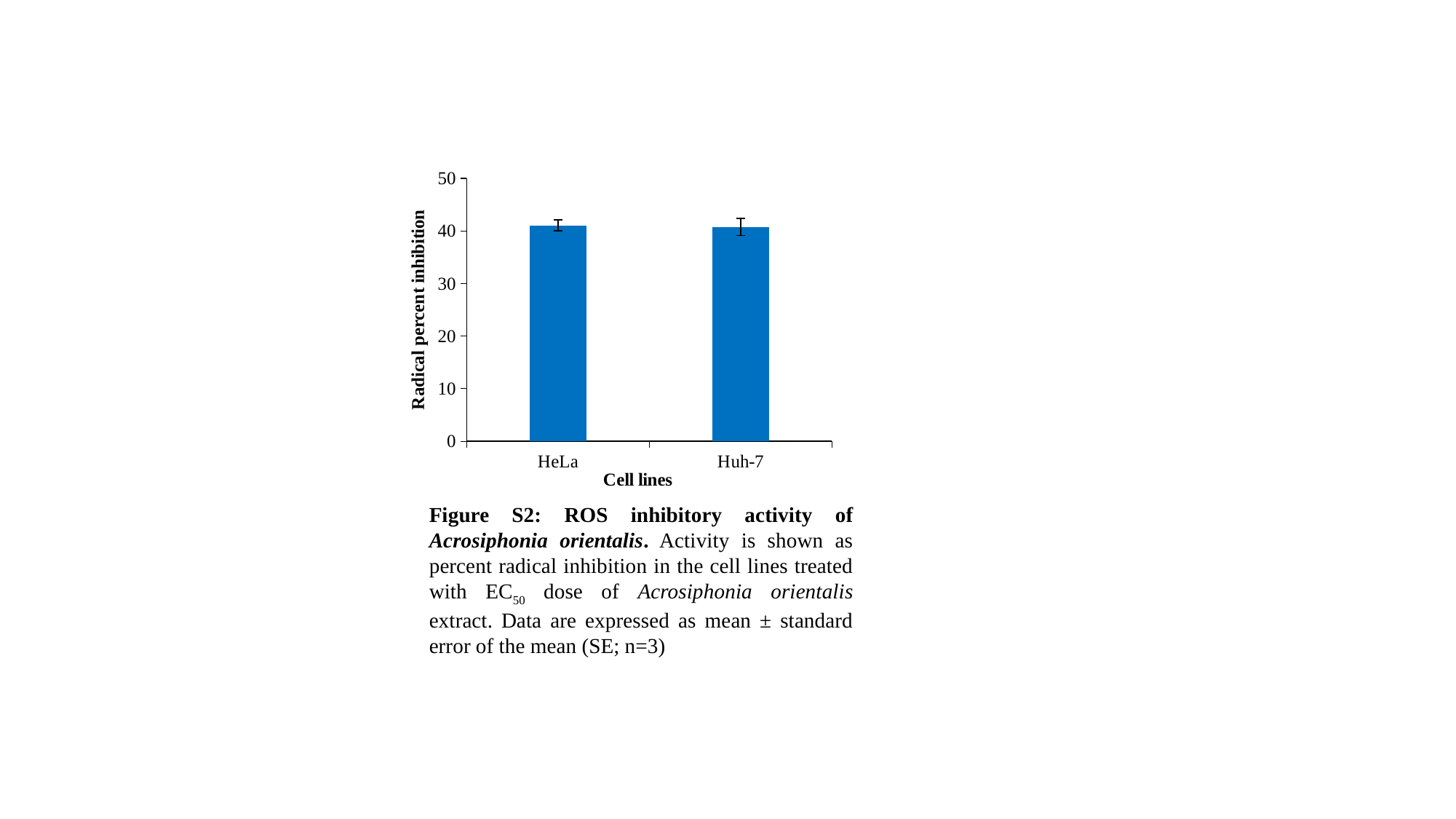

### Chart
| Category | |
|---|---|
| HeLa | 41.05375358448999 |
| Huh-7 | 40.75715935500677 |Figure S2: ROS inhibitory activity of Acrosiphonia orientalis. Activity is shown as percent radical inhibition in the cell lines treated with EC50 dose of Acrosiphonia orientalis extract. Data are expressed as mean ± standard error of the mean (SE; n=3)

## Slide 3
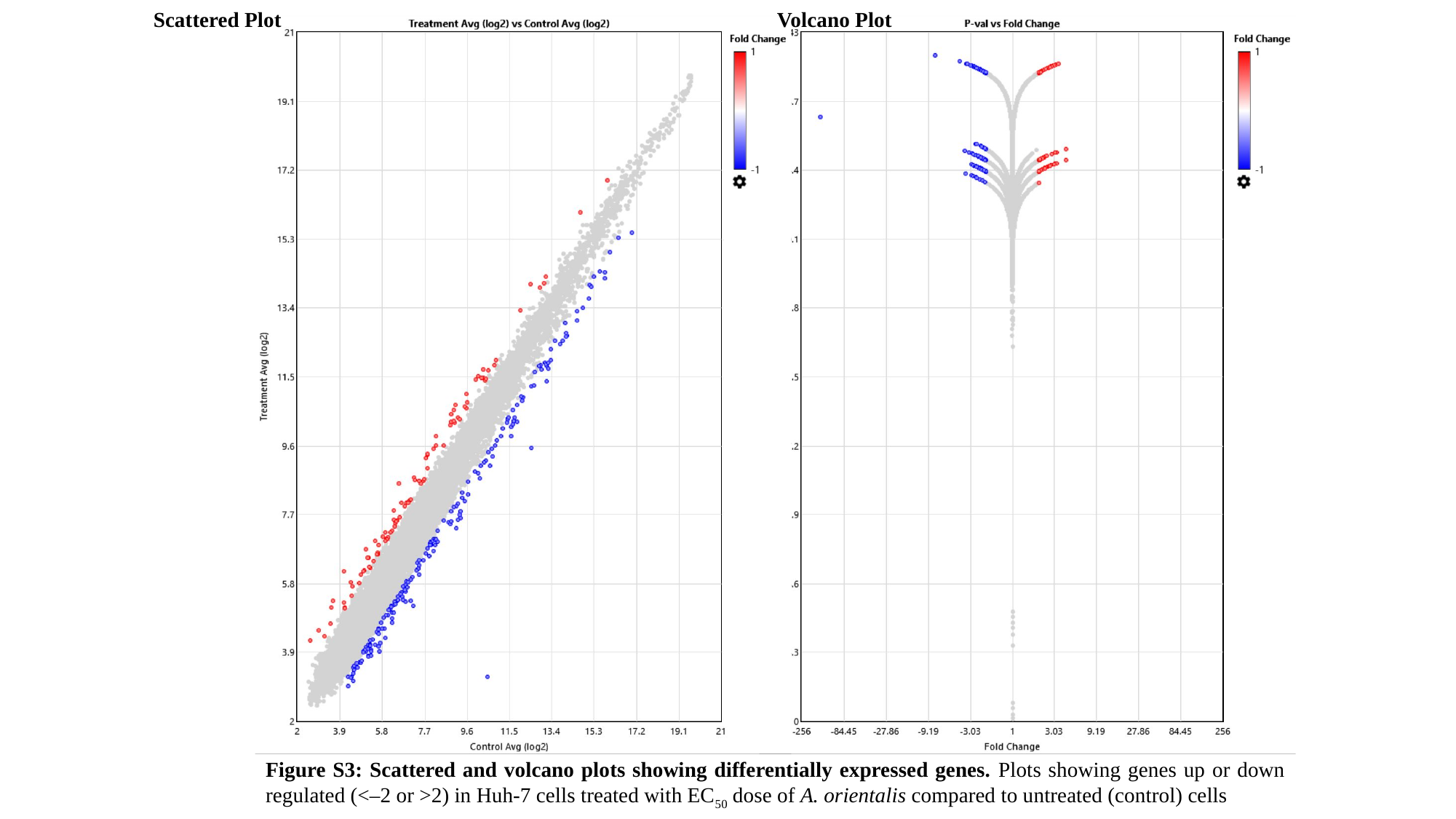

Scattered Plot Volcano Plot
Figure S3: Scattered and volcano plots showing differentially expressed genes. Plots showing genes up or down regulated (<–2 or >2) in Huh-7 cells treated with EC50 dose of A. orientalis compared to untreated (control) cells

## Slide 4
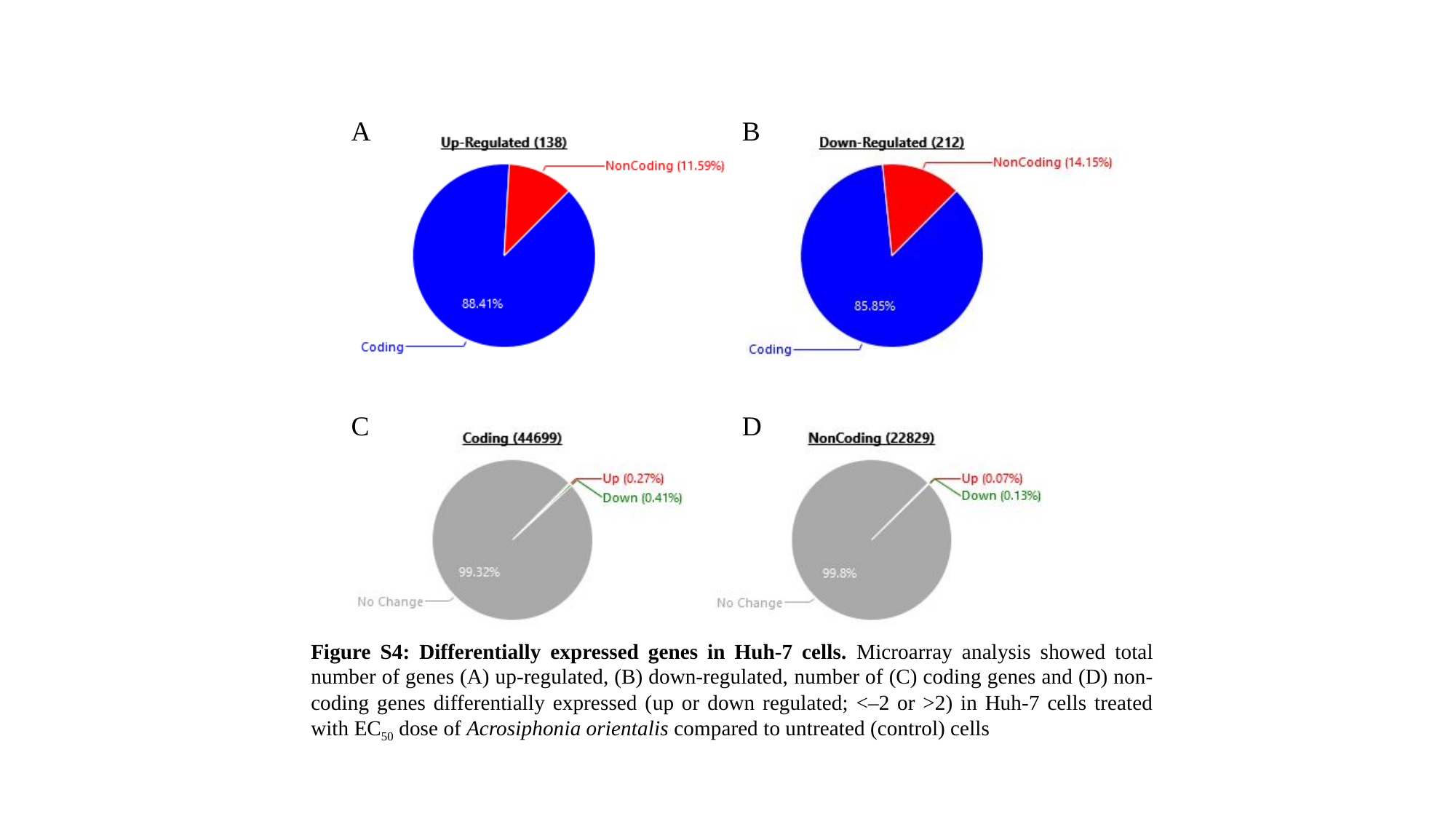

A B
C D
Figure S4: Differentially expressed genes in Huh-7 cells. Microarray analysis showed total number of genes (A) up-regulated, (B) down-regulated, number of (C) coding genes and (D) non-coding genes differentially expressed (up or down regulated; <–2 or >2) in Huh-7 cells treated with EC50 dose of Acrosiphonia orientalis compared to untreated (control) cells

## Slide 5
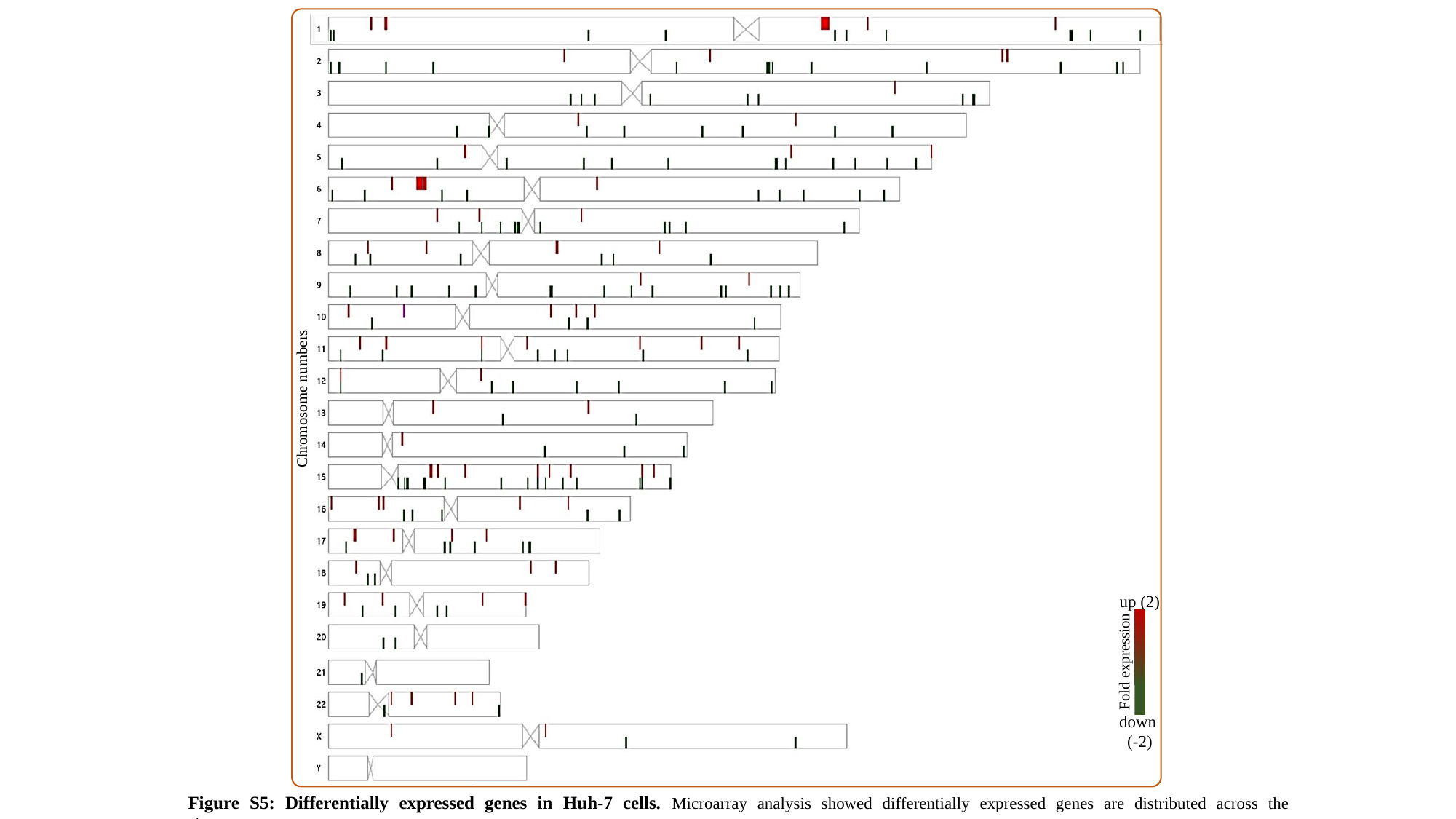

up (2)
down
(-2)
Chromosome numbers
Fold expression
Figure S5: Differentially expressed genes in Huh-7 cells. Microarray analysis showed differentially expressed genes are distributed across the chromosomes.

## Slide 6
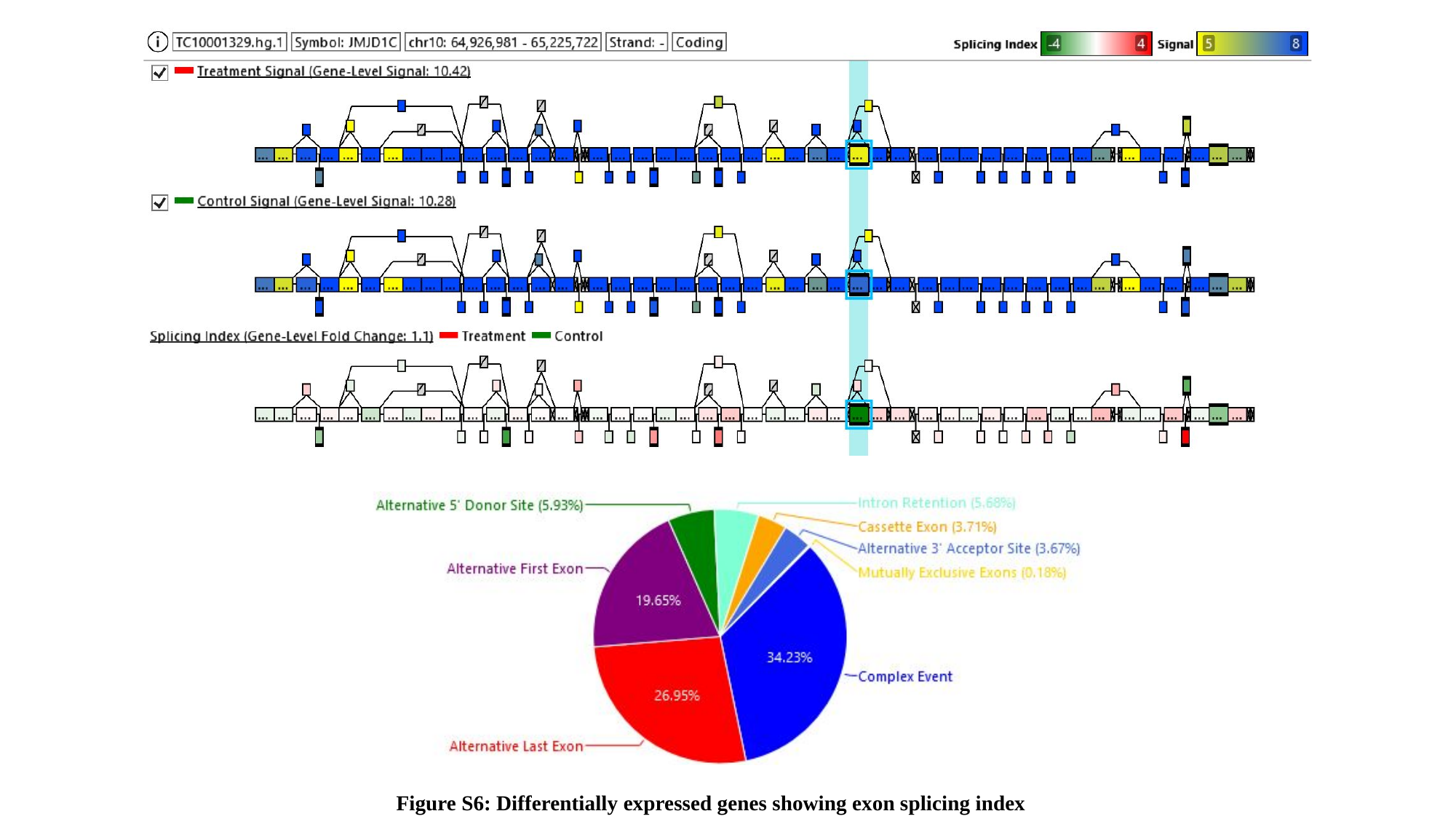

Figure S6: Differentially expressed genes showing exon splicing index
