# Supplementary material for: Metabolite Profiling, Biological and Molecular Analyses Validate the Nutraceutical Potential of Green Seaweed Acrosiphonia orientalis for Human Health
Source: Nutrients. 2024 Apr 19;16(8):1222. doi: 10.3390/nu16081222 (PMC11055090; doi:10.3390/nu16081222)
Supplement: Supplementary file 1 [file nutrients-16-01222-s001.zip › Table S1.pdf]

Table S1: Primer sets and optimized PCR conditions for quantitative real-time PCR (Tanna et al., 2020, *Molecular Biology Reports*. 47, 7403–7411) [26].

<table>
<tr><th rowspan="2">S. No.</th><th rowspan="2">Genes</th><th colspan="2">Primer pair (5′ → 3′)</th><th rowspan="2">qRT-PCR condition</th></tr>
<tr><td>Forward</td><td>Revers</td></tr>
<tr><td>1.</td><td>GAPDH</td><td>CAAGGTCATCCATGACAACTTTG</td><td>GTCCACCACCCTGTTGCTGTAG</td><td rowspan="6">Initial denaturation:
94 °C for 5 min

40 cycles:
94 °C- 30 s
58 °C- 45 s
72 °C- 60 s

Melt curve:
50 to 94 with 0.05°C</td></tr>
<tr><td>2.</td><td>Cas3</td><td>ATGGAAGCGAATCAATGGAC</td><td>GCCATGTCATCATCAACACC</td></tr>
<tr><td>3.</td><td>p53</td><td>ATGGCCATCTACAAGCAG</td><td>ACAGTCAAGAGCCAACCTCAG</td></tr>
<tr><td>4.</td><td>DNMT</td><td>AGAACGCCTTTAAGCGCCG</td><td>CCGTCCACTGCCACCAAAT</td></tr>
<tr><td>5.</td><td>CDC2</td><td>ACAGGTCAAGTGGTAGCCATGA</td><td>ACCTGGAATCCTGCATAAGCA</td></tr>
<tr><td>6.</td><td>BAX</td><td>GTGGCAGCTGACATGTTTTC</td><td>GGAGGAAGTCCAATGTCCAG</td></tr>
</table>
