# Supplementary material for: Metabolite Profiling, Biological and Molecular Analyses Validate the Nutraceutical Potential of Green Seaweed Acrosiphonia orientalis for Human Health
Source: Nutrients. 2024 Apr 19;16(8):1222. doi: 10.3390/nu16081222 (PMC11055090; doi:10.3390/nu16081222)
Supplement: Supplementary file 1 [file nutrients-16-01222-s001.zip › Table S3.pdf]

Table S3: Half maximal effective concentration (EC_{50}) dose of *Acrosiphonia orientalis* for different bioactivities

**Bioactivities**	**EC_{50} dose^**
Total antioxidant	40 ± 1
Radical scavenging	25 ± 7
Reducing capacity	5 ± 1
Anti-proliferative (HeLa)	20 ± 2
Anti-proliferative (Huh7)	25 ± 1

^Dose (mean ± SE; n=3) in mg Dw (dry weight)

Values are round-up as per significant certainty numbers
