# Supplementary material for: Metabolite Profiling, Biological and Molecular Analyses Validate the Nutraceutical Potential of Green Seaweed Acrosiphonia orientalis for Human Health
Source: Nutrients. 2024 Apr 19;16(8):1222. doi: 10.3390/nu16081222 (PMC11055090; doi:10.3390/nu16081222)
Supplement: Supplementary file 1 [file nutrients-16-01222-s001.zip › Table S4.pdf]

Table S4: Correlation matrix of different activities (total antioxidant, scavenging and reducing) and phenolic (TPC) and flavonoid (TFC) contents of *Acrosiphonia orientalis*

Correlation matrix (Pearson):

Variables	TPC	ABTS	DPPH	RP	TFC
TPC	**1**	**0.916**	**0.975**	0.670	**0.949**
ABTS	**0.916**	**1**	**0.913**	0.867	**0.983**
DPPH	**0.975**	**0.913**	**1**	0.630	**0.969**
RP	0.670	0.867	0.630	**1**	0.775
TFC	**0.949**	**0.983**	**0.969**	0.775	**1**

Values in bold are different from 0 with a significance level alpha=0.05

p-values (Pearson):

Variables	TPC	ABTS	DPPH	RP	TFC
TPC	**0**	**0.029**	**0.005**	0.216	**0.014**
ABTS	**0.029**	**0**	**0.031**	0.057	**0.003**
DPPH	**0.005**	**0.031**	**0**	0.255	**0.006**
RP	0.216	0.057	0.255	**0**	0.123
TFC	**0.014**	**0.003**	**0.006**	0.123	**0**

Coefficients of determination (Pearson):

Variables	TPC	ABTS	DPPH	RP	TFC
TPC	**1**	0.839	0.951	0.449	0.901
ABTS	0.839	**1**	0.833	0.752	0.966
DPPH	0.951	0.833	**1**	0.397	0.939
RP	0.449	0.752	0.397	**1**	0.601
TFC	0.901	0.966	0.939	0.601	**1**

TPC: Total Phenolic Content; ABTS: Total antioxidant activity; DPPH: Radical Scavenging Activity; RP: Reducing Power, and TFC: Total Flavonoid Content
